# Brain atrophy in the visual cortex and thalamus induced by severe stress in animal model

**DOI:** 10.1038/s41598-017-12917-z

**Published:** 2017-10-06

**Authors:** Takanobu Yoshii, Naoya Oishi, Kazuya Ikoma, Isao Nishimura, Yuki Sakai, Kenichi Matsuda, Shunji Yamada, Masaki Tanaka, Mitsuhiro Kawata, Jin Narumoto, Kenji Fukui

**Affiliations:** 1 Department of Psychiatry, Graduate School of Medical Science, Kyoto Prefectural University of Medicine, 465 Kajii-cho, Kamigyo-ku, Kyoto, 602-8566 Japan; 2 Kyoto Prefectural Rehabilitation Hospital for Mentally and Physically Disabled, Naka-ashihara, Joyo-city, Kyoto 610-0113 Japan; 3 Research and Educational Unit of Leaders for Integrated Medical System, Center for the Promotion of Interdisciplinary Education and Research, Kyoto University, 54 Kawahara-cho, Sakyo-ku, Kyoto, 606-8507 Japan; 4 Department of Orthopaedics, Graduate School of Medical Science, Kyoto Prefectural University of Medicine, 465 Kajii-cho, Kamigyo-ku, Kyoto, 602-8566 Japan; 5 Kawagoe Hospital, 48 Jyodojibanba-cho, Sakyo-ku, Kyoto, 606-8412 Japan; 6 Department of Neural Computation for Decision-making, ATR Brain Information Communication Research Laboratory Group, 2-2-2 Hikaridai, Seika-cho, Soraku-gun, Kyoto, 619-0288 Japan; 7 Department of Anatomy and Neurobiology, Graduate School of Medical Science, Kyoto Prefectural University of Medicine, 465 Kajii-cho, Kamigyo-ku, Kyoto, 602-8566 Japan; 8 School of Health Sciences, Bukkyo University, 96 Hananobo-cho, Kita-ku, Kyoto, 603-8301 Japan; 9 Health Care Center, Kyoto Prefectural University of Medicine, 465 Kajii-cho, Kamigyo-ku, Kyoto, 602-8566 Japan

## Abstract

Psychological stress induces many diseases including post-traumatic stress disorder (PTSD); however, the causal relationship between stress and brain atrophy has not been clarified. Applying single-prolonged stress (SPS) to explore the global effect of severe stress, we performed brain magnetic resonance imaging (MRI) acquisition and Voxel-based morphometry (VBM). Significant atrophy was detected in the bilateral thalamus and right visual cortex. Fluorescent immunohistochemistry for Iba-1 as the marker of activated microglia indicates regional microglial activation as stress-reaction in these atrophic areas. These data certify the impact of severe psychological stress on the atrophy of the visual cortex and the thalamus. Unexpectedly, these results are similar to chronic neuropathic pain rather than PTSD clinical research. We believe that some sensitisation mechanism from severe stress-induced atrophy in the visual cortex and thalamus, and the functional defect of the visual system may be a potential therapeutic target for stress-related diseases.

## Introduction

It has been shown that stress causes many diseases and pains. In particular, post-traumatic stress disorder (PTSD) is one of the most common mental disorders with a high lifetime prevalence of approximately 6–10%^1,2^. Although the stress-disease relationship has been estimated in PTSD pathogenesis, the prevalence of PTSD in trauma-exposed humans is only approximately 20% (male: 15%; female: 25%)^3^. According to previous neuroimaging studies of PTSD, the atrophic areas appear to vary between studies. These areas include the hippocampus, anterior cingulate cortex (ACC), posterior cingulate cortex^4^, insular cortex^5^, orbitofrontal cortex^6^, ventromedial prefrontal cortex^7^, occipital cortex^8^, calcarine sulcus^9^, and amygdala^10^. Neuropathic pain brings stress, and central sensitisation from pain has been shown to be one of the important mechanisms. A recent meta-analysis in chronic neuropathic pain has shown decreased grey matter volume in the bilateral anterior insula and thalamus, as well as the right superior frontal gyrus and left post central gyrus^11^. However, it is not yet clear whether such atrophy is caused by sensitisation from neuropathy, psychological stress from chronic pain, or stress from the environmental aspects of patients. Although many studies of stress have been reported, it has been unclear whether these inconsistent results were due to stress.

Past clinical studies in PTSD have attempted to clarify the relationship between stress and atrophy. Kasai *et al*. reported that the atrophy in ACC is related to stress when comparing trauma in exposed and non-exposed twins^12^. A recent longitudinal brain magnetic resonance imaging (MRI) study on victims of the Great East Japan earthquake revealed that decreased grey matter volume in the left orbitofrontal cortex is negatively associated with PTSD symptoms^13^. A voxel-wise meta-analysis of grey matter changes in individuals with PTSD was also conducted, and occipital regions were proposed as the “stress related” atrophic area^14^. Since it is difficult to omit all confounding factors of stress in human research, such as stress paradigm, patient backgrounds, and the genetic dispositions of subjects, we believe that the causal relationship between stress and atrophy has not been adequately verified. Therefore, we conducted an animal model study to verify the relationship between stress and atrophy.

Single-prolonged stress (SPS)^15^ represents one of the rodent models of PTSD stress model, which has demonstrated key phenotypic characteristics^16–19^ of PTSD. The authors have also reported that SPS induces adrenocorticotrophic hormone (ACTH) depletion in the pituitary and alters vasopressin systems^20^ and consider this model to be simple and reproducible. However, a comprehensive and global brain analysis of SPS has rarely been described.

Voxel-based morphometry (VBM)^21^ is a comprehensive statistical analysis to assess regional morphological differences. Since a global analysis of whole brain morphology in animals is difficult, we have adopted this method. We expected VBM, as applied in this animal study, to enable us to bridge the gap between animal stress research and pathophysiological study with neuroimaging in humans.

Microglia is widely distributed in the whole brain, and stress-induced microglial activation due to acute water-immersion restraint stress^22^, chronic stress^23^, and prenatal stress^24^ has been reported in past research. Therefore, we assumed that microglial activation would occur as a stress reaction in the stress-related region in the brain. The relationship between atrophy and neuroinflammation in neurodegenerative diseases has also been described^25^, and it has been hypothesised that altered microglial functions could lead to neuropsychiatric disorders^26^. However, it has been demonstrated that reactive astrocytes could contribute to the signal in addition to reactive microglia in PK11195 [N-methyl - N-(1-methylpropyl)-1-(2-chlorophenyl) – isoquinoline - 3-carboxamide] PET imaging^27^. Although the study of microglia in humans has been difficult, cytokines have been well investigated. Clinical PTSD research seems to have developed primarily due to the demands of the military, and autopsy research in human PTSD without complication of traumatic brain injury has rarely been reported. Therefore, there is still a need for animal studies to clarify the relationship between microglial activation and stress-related diseases.

Accordingly, we conducted an animal VBM study to verify the relationship between severe stress and atrophy. We also hypothesised that microglial activation occurs in the atrophic brain sites, which were obtained in this VBM study.

## Results

### Whole-brain Voxel-based morphometry (VBM)

**Figure 1:**
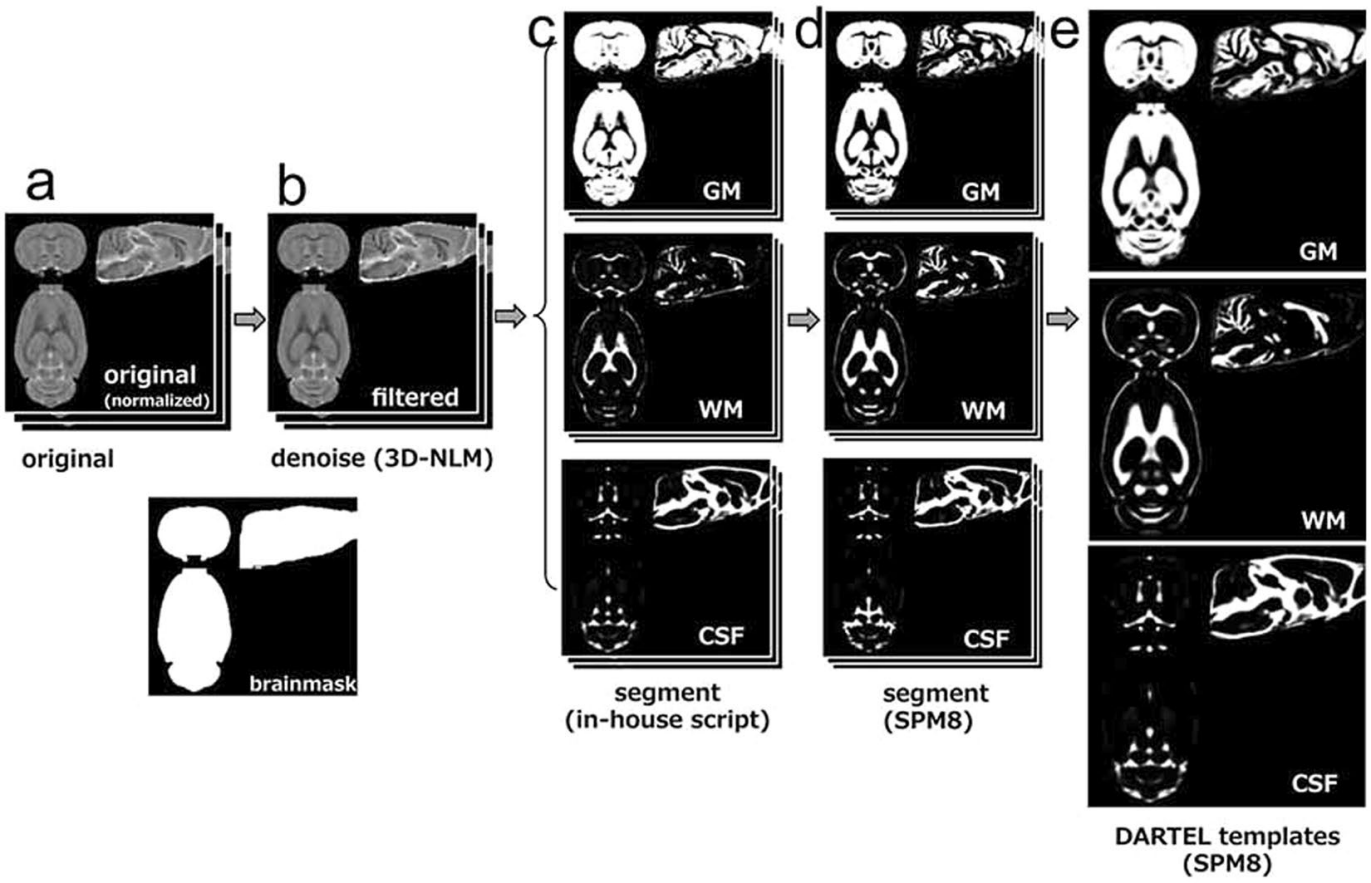
Manufacturing anatomical template. In-house experimental rat brain templates for a T2-weighted image and probabilistic maps of grey matter (GM), white matter (WM), cerebrospinal fluid (CSF), and its manufacturing course; (**a**) the original images, (**b**) the de-noised images by an in-house three-dimensional non-local means (3D-NLM) filter script, (**c**) the segmented images by an in-house Matlab script, (**d**) the segmented images customised by the SPM8 tool for a diffeomorphic anatomical registration using exponentiated lie algebra (DARTEL) template creation, (**e**) the manufactured DARTEL template.

**Figure 2:**
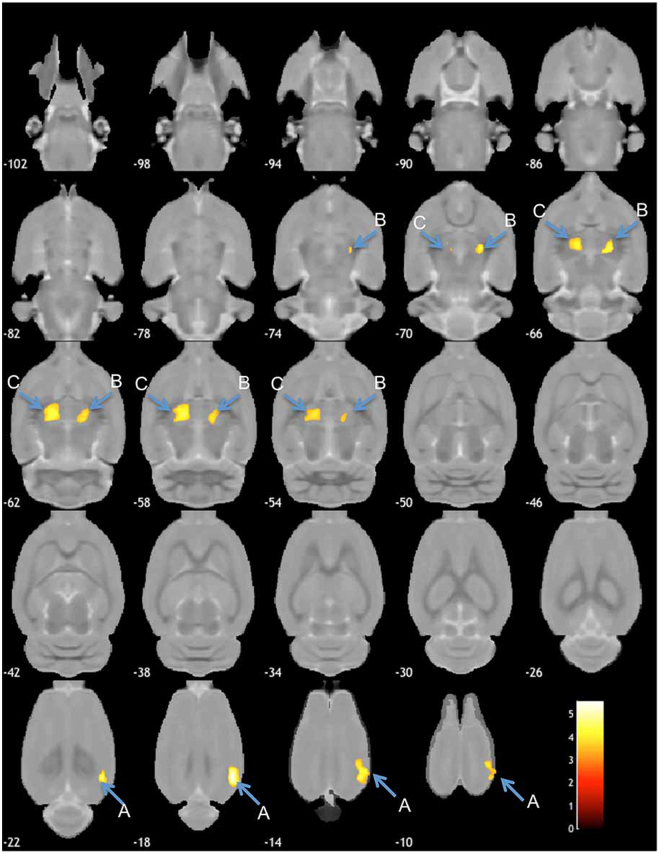
Results of global VBM detecting brain atrophy in axial slices. Axial brain slices showing a significant atrophic effect of SPS in the part of the sensory cortex (cluster **A**), and the bilateral thalamus (clusters **B** and **C**), respectively (SPS, n = 18; sham, n = 17; height level *p* < 0.001, cluster-FWE corrected *p* < 0.05). Colour bar units refer to t-scores. In cluster A, the peak coordinate exists in the right primary visual cortex (V1) and the cluster extends to the right secondary visual (V2) cortex. Both clusters in the bilateral thalamus (clusters **B** and **C**) extend to the ventral part of the thalamus to the dorsal medial side. In addition, cluster C extends to the dorsal lateral side of the left thalamus. We consider that clusters B and C do not include the posterior lateral nuclei and the lateral geniculate nuclei in the thalamus.

**Figure 3:**
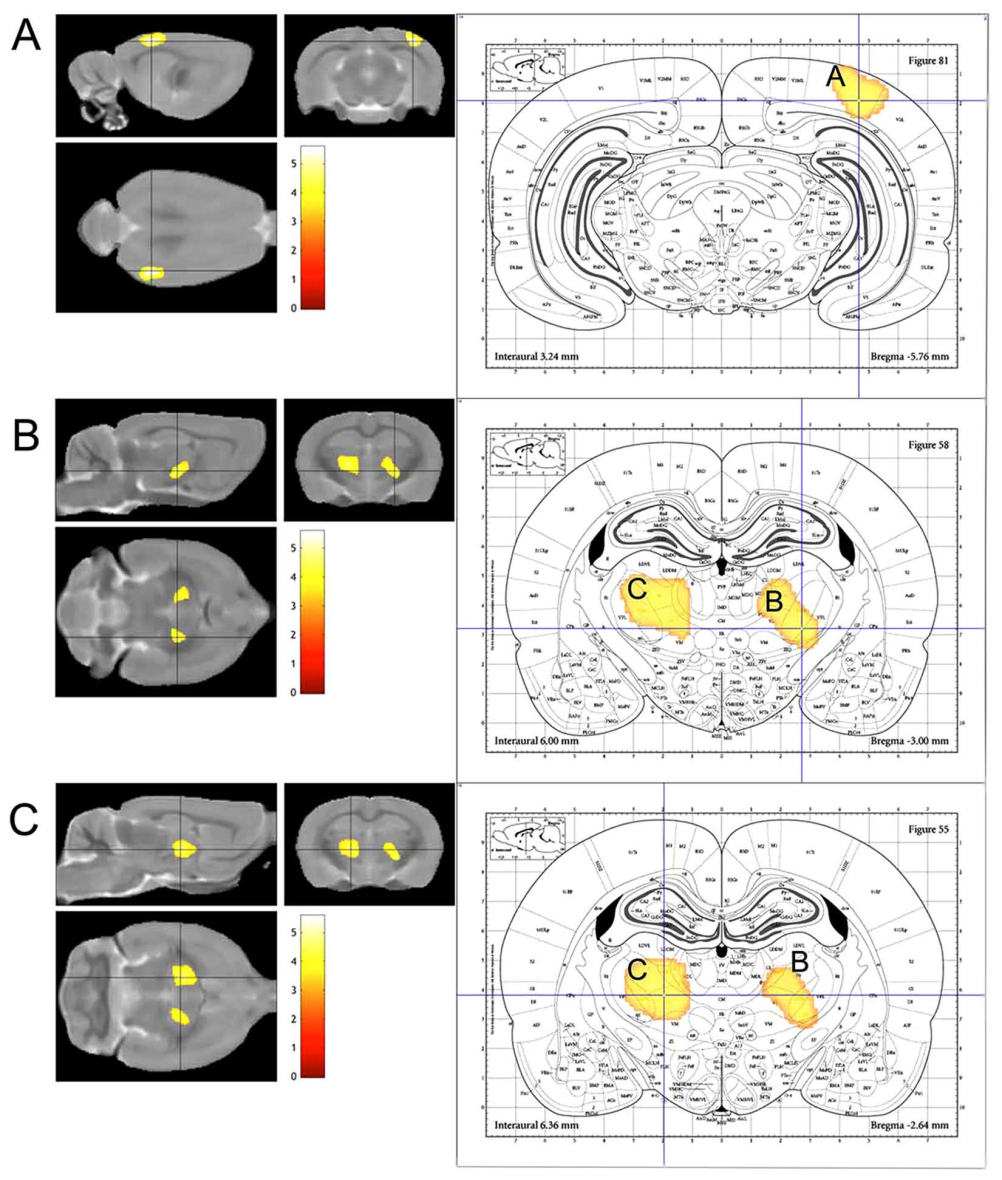
Peak coordinate of atrophic brain clusters showing a significant effect. Three-dimensional brain sections showing a significant atrophic effect of SPS in the part of the sensory cortex (cluster **A**), and the thalamus (clusters **B** and **C**), respectively (SPS, n = 18; sham, n = 17; height level *p* < 0.001, cluster-FWE corrected *p* < 0.05). Coronal sections with the peak coordinate piled upon the rat brain atlas^68^ are also presented. Cross hair lines indicate the peak coordinate. Colour bar units refer to t-scores. According to the rat brain atlas, the peak coordinate of cluster **A** exists in the right primary visual area. The peak coordinate of cluster **B** is located in the right ventral posteromedial nucleus in the thalamus. The peak coordinate of cluster **C** exists in the left ventral lateral nucleus of the thalamus.

**Figure 4:**
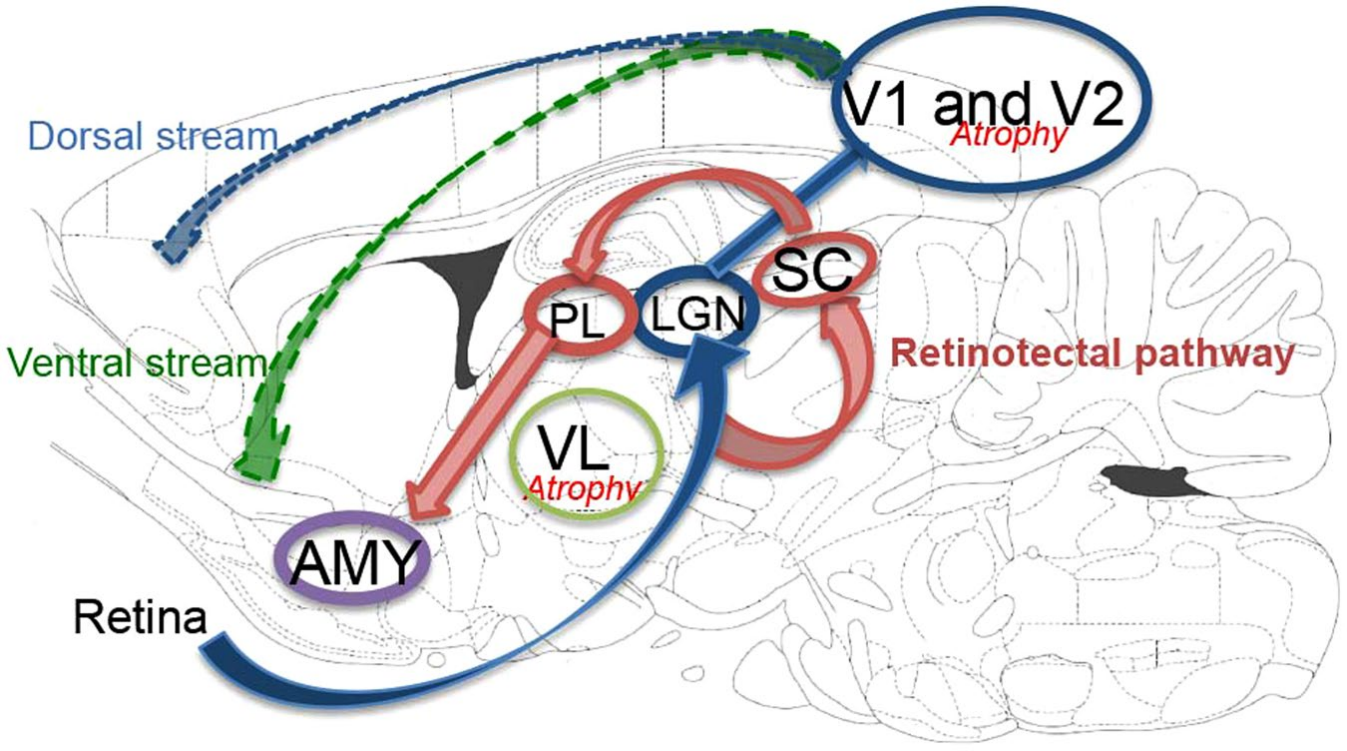
The schematic summary of results in VBM piled upon the visual stream. The schema of results piled upon the visual stream and the retinotectal pathway in the sagittal sections of the rat brain atlas^68^. The information from the retina projects to the lateral geniculate nucleus of the thalamus (LGN) and the superior coliculus (SC). The retinotectal pathway: the projection from the superior colliculus to the amygdala (AMY) via the posterior lateral nucleus of the thalamus (PL)^32^ and the cortical-cortical long-range fasciculi in the ventral stream to the limbic areas^55^ including the amygdala play key roles in fast visual fear response. Our obtained result shows atrophy in the visual cortex (V1 and V2), and in the ventral lateral area in the thalamus (VL). Because of the atrophy in the visual cortex, the contribution of the retinotectal pathway to visual processing might have relatively increased.

**Figure 5:**
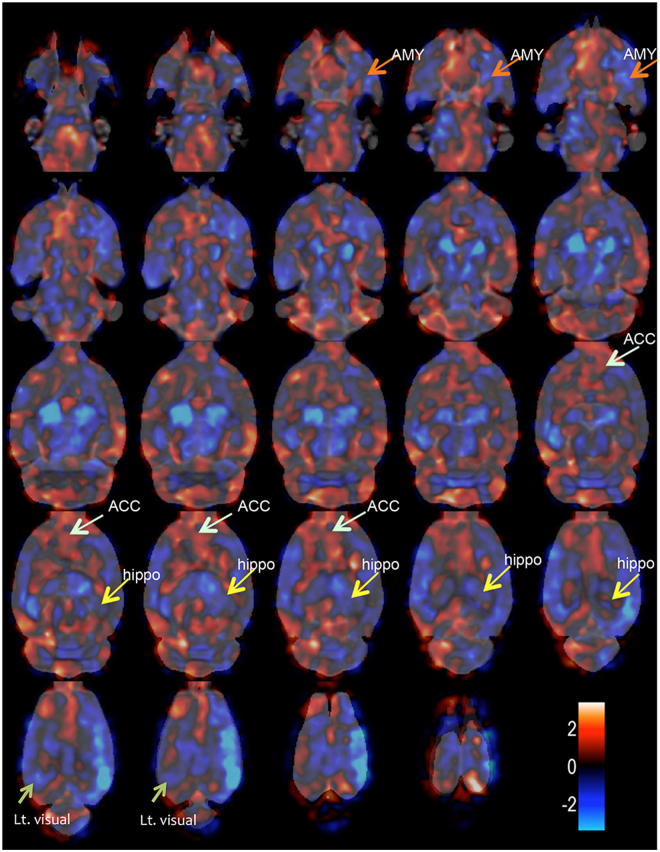
T-score map for the VBM result. T-score distribution map of axial brain slices (SPS, n = 18; sham, n = 17). The blue colour indicates atrophy, and the red colour refers to hypertrophy. It has been suggested that fear learning is related to areas including the hippocampus (Hippo), amygdala (AMY), and ACC. In this t-score map, both the hippocampus and amygdala seem to indicate atrophic trends; however, these did not reach significance. In contrast, ACC, indicates a hypertrophic trend without reaching significance. Although we detected significant atrophy in the right visual cortex, the left visual cortex (Lt. visual) reflected only an atrophic trend, but did not reach significance.

We considered that VBM is an appropriate approach to avoid the experimenter bias on this animal study. We therefore conducted VBM for a global and comprehensive brain analysis to clarify the effect of SPS. Animals were exposed to SPS or sham stress (ether anaesthesia only), and after these stresses, they were maintained in cages for 7 days. Then, they were perfused and fixed with 4% paraformaldehyde. We obtained brains with skulls and immersed them in the same fixative for 3 days. MRI acquisition was carried out using the T2-weighted three-dimensional (3D) protocol (mentioned in the *Ex-vivo* brain MRI section). The experimental brain MR images were segmented into grey matter and spatially normalised using diffeomorphic anatomical registration using exponentiated lie algebra (DARTEL) in statistical parametric mapping software (SPM8) with the in-house SD rat template (Fig. 1). VBM was then carried out (SPS: n = 18; sham: n = 17). The VBM result of the axial slices of the whole brain is shown in Fig. 2, and the peak coordinate of clusters is presented in Fig. 3. VBM revealed three significant atrophic clusters induced by SPS in the areas of the right visual cortex (cluster A in Figs 2 and 3) and the bilateral thalamus (clusters B and C in Figs 2 and 3), respectively; SPS: n = 18, sham: n = 17, cluster-FWE corrected *p* < 0.05 (Table 1). In cluster A, the peak coordinate is in the primary visual cortex and the cluster extends from the primary visual area. In clusters B and C, the bilateral thalamus is detected and both clusters seem to extend to the ventral-lateral area in the thalamus. We piled these atrophic areas upon the visual stream and the retinotectal pathway (Fig. 4) and considered that cluster A is subject to the visual stream; however, neither the posterior lateral nucleus in the thalamus nor the lateral geniculate nucleus reaches significance. Surprisingly, we did not detect significant atrophic clusters in the hippocampus, amygdala, and ACC—areas that have previously been identified as contributors to PTSD pathogenesis^28^ (Fig. 5). On the other hand, we did not detect hypertrophy induced by SPS in this statistical threshold. However, moderate threshold (*p* < 0.001, uncorrected) allowed us to detect a hypertrophic trend in a cluster extending from the medial part of the secondary visual cortex (cluster D in Supplementary Fig. 1 and Supplementary Table).

**Table 1:** Atrophic clusters showing significant effects between the SPS and sham groups in voxel-based morphometry.

	Cluster	Cluster	Peak	Peak	Peak	Estimated area (peak coordinate)
	*p* (FWE-corrected)	Cluster size (voxels)	T value	Z value	*p* (uncorrected)	
Cluster A	0.003	1360	5.57	4.64	<0.001	Visual cortex (rt V1)
Cluster B	0.019	960	4.64	4.04	<0.001	Thalamus (rt VPM)
Cluster C	0.001	1825	4.53	3.97	<0.001	Thalamus (lt VL)

rt: right, lt: left, V1: primary visual cortex, VPM: ventral posteromedial thalamic nucleus, VL: ventral lateral thalamic nucleus.

### Immunohistochemistry for lba-1

**Figure 6:**
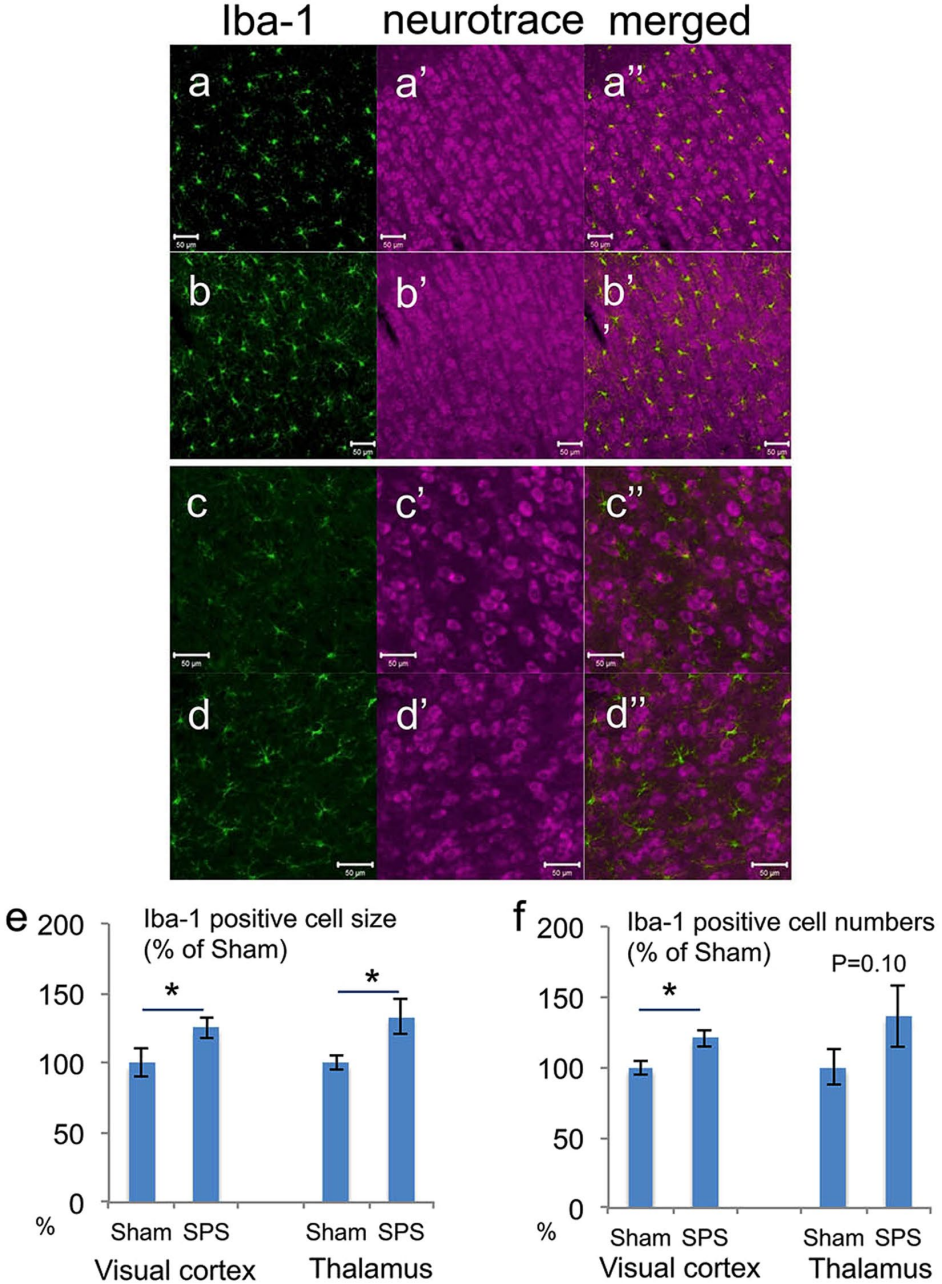
Immunohistochemistry for IBa-1 as microglial activation and the result of morphometry. Representative photomicrographs of Iba-1 immunofluorescence in the visual cortex (a: sham, n = 6 and b: SPS, n = 5) and thalamus (c: sham, n = 6 and d: SPS, n = 5) with counter stain of neurotrace (**a’**, **b’**, **c’**, and **d’**) and merged (**a”**, **b”**, **c”**, and **d”**) on the 7^th^ day after the stress exposure and the morphometry result (**e** and **f**). SPS-exposed rats showed enhanced Iba-1 IR and dendritic branch compared with sham-stressed rats both in the visual cortex and the thalamus (**a** and **b**). The statistical analysis showed significant hypertrophy in the visual cortex and the thalamus (**e**). A significant increase in the number of Iba-1-positive cells was also detected only in the visual cortex (**f**). The number of Iba-1-positive cells in the thalamus failed to reach significance but indicated an increasing trend (*p* = 0.10, one-tailed Welch’s t-test) (**f**). *Indicates *p* < 0.05 on one-tailed Welch’s t-test.

Microglia is distributed in the whole brain and is activated by stress. In order to confirm the regional stress reaction, we investigated lba-1-immunoreactivity (IR) as an activated microglia marker in the right thalamus and visual cortex, which contained the atrophic sites in this VBM analysis. In the visual cortex, we observed that the increased number of IBa-1 positive cells enhanced Iba-1 IR (Fig. 6a,b). Statistical analysis showed that not only the 25.5% larger size (SPS: n = 5; sham: n = 6; t = 2.638, *p* = 0.028*, one-tailed Welch’s t-test, Fig. 6e) but also the 20.8% increased number (SPS: n = 5; sham: n = 6; t = 2.073, *p* = 0.038*, one-tailed Welch’s t-test, Fig. 2f) of Iba-1 positive cells in the visual cortex was detected. In the thalamus, we observed that enhanced Iba-1 IR (Fig. 6c,d) and morphometry revealed 33.3% hypertrophy (SPS: n = 5; sham: n = 6; t = −2.282, *p* = 0.039*, one-tailed Welch’s t-test, Fig. 2e) in the Iba-1-positive cells and an increase in the number of Iba-1-positive cells, indicating an increasing trend (SPS: n = 5; sham: n = 6; t = −1.421, *p* = 0.099, one-tailed Welch’s t-test, Fig. 6f).

## Discussion

Severe stress resulted in atrophy in areas of the thalamus and visual cortex, as well as the enhancement of Iba-1-IR in these areas, indicating the activation of the microglia. In this study, there are almost no confounding factors for atrophy, and the relationship between atrophy and stress must be rigid. The relationship between brain atrophy and microglial activation was also demonstrated; thus, we showed that severe stress could cause sensitisation in the visual cortex and the thalamus, both at the microscopic level and at the macroscopic level of brain atrophy detection using MRI. Interestingly, atrophy in the bilateral thalamus and pain-related sensory areas have been reported in chronic pain studies. However, our results in VBM have more similarities to studies on chronic pain than to studies on PTSD. Although the relationship between PTSD pathogenesis and fear-related circuits including the amygdala, hippocampus and ACC has been discussed^28^, we were not able to confirm the relationship between stress and atrophy in these sites.

This is the first study to reveal stress-induced regional brain atrophy in the bilateral thalamus in rodents. Although animal VBM analysis has not been conducted in pain research, a recent meta-analysis of chronic neuropathic pain in humans suggested consistent decreased grey matter in the bilateral thalamus^11^. Amazingly, our results showed atrophy in the bilateral thalamus via 1-day severe psychological stress without a neuropathic pain paradigm. Our results suggest that sensitisation from neuropathic pain may not be a requirement for brain atrophy in chronic pain. Indeed, chronic pain is contingent in psychological toll or torture^29^, and our result may show one of the central mechanisms through which severe stress influences the pain threshold. On the other hand, it has also been reported that people with higher re-experiencing scores tended to have decreased thalamus volume^30^, although atrophy of the thalamus has rarely been reported in human PTSD. Atrophy in the pulvinar nucleus of thalamus in trauma survivors without PTSD has been reported^31^. An animal study demonstrated that optgenetic activation on the superior colliculus projecting to the posterior lateral nucleus of the thalamus, which is pulvinar nucleus in humans, could mimic visually evoked fear behavioral response^32^, which indicates that the retinotectal pathway plays key roles in fear behavioral response. In our result, the visual thalamic nuclei including the posterior lateral nuclei and the lateral geniculate nuclei seem to be out of the atrophic regions obtained in the VBM, and the visual fast fear response via the retinotectal pathways was not disrupted.

This is also the first study to show that severe stress directly induces atrophy in visual areas extending from the primary visual cortex in rodents. The results are also similar to those of human pain studies, as it has been reported that the chronic neuropathic pain of a spinal cord injury induced atrophy in pain-related primary sensory and visual-related regions^33^. On the other hand, a meta-analysis review for PTSD suggested that atrophy in occipital regions is stress-related^14^, and reduced visual cortex grey matter volume has been reported in subjects who experienced childhood sexual abuse^34^ and subjects who witnessed domestic violence during childhood^35^. It has been discussed that parental verbal abuse, the witnessing of domestic violence, and sexual abuse appear to specifically target brain regions (the auditory, visual and somatosensory cortex) and pathways that process and convey the aversive experience^36^. Indeed, aversive learning mediates the orientation selectivity of the neuronal population in the visual cortex^37^. It has also been reported that hyperactivity in the visual cortex in PTSD patients was observed using resting-state functional MRI^38^. We therefore consider that some sensitisation mechanism from severe stress might have brought atrophy in the visual cortex. In addition, we believe that the contributions of the retinotectal pathways to visual processing would increase, because of the atrophy in the visual cortex obtained by the VBM.

In our study, only right visual cortex was detected in the global VBM. However, according to the t-score map in our VBM study, we consider that left visual cortex indicates an atrophic trend. In order to achieve enough quality in MRI acquisition for VBM analysis, we applied the *ex-vivo* protocol. Because of this protocol, we cannot completely rule out the influence of fixation on atrophy in the left visual cortex induced by severe stress. We consider that further research on laterality should be conducted in a separate research project.

Interestingly, recent research has shown that limbic areas are functionally decoupled and that the visual cortex has a central role in the fear conditioning paradigm in humans^39^. Koizumi *et al*. reported that fear can be reduced without decreasing amygdala activation using non-explicit fear exposure with functional MRI (decoded neuro-feedback)^40^. It has also been demonstrated that applying rhythmic transcranial magnetic stimulation (rTMS) to the visual cortex leads to decreased episodic memory retrieval in the visual field contralateral to the site of stimulation^41^. In animals, it has been reported that the primary visual cortex indicates reward-timing activity^42^ and that the secondary visual cortex contributes to object recognition memory^43^. Our obtained results contain the secondary visual cortex, and align with the report about impairments to object novelty recognition by SPS^44^. Although the visual cortex in rodents has a simpler structure than in humans, the fast response mechanism of the retinotectal pathways and the reward-timing activity of the visual cortex in rodents enable us to discuss and translate the relationship between vision and fear from rodents to humans. Together, there is a synchronism among the research of the visual cortex pertaining to stress-induced atrophy. Our results, involving neuronal activation by the fear conditioning paradigm^39^ and the improvement of fear via functional modulation^40^, align with those of previous research. We believe that the approaches targeting the visual area for both fear and aversive memory reduction without explicit counter-conditioning fear stimulation would be promising interventions for fear-related syndromes.

This is the first study to explore microglial activation in the obtained stress-induced brain atrophic sites and to verify a triangular relationship among stress, neuroinflammation, and atrophy. We found a stress effect in the atrophic areas through VBM. Since a recent study demonstrated that flicker light stimulation modulates the microglia in the visual cortex^45^, we considered that microglial activation indicates sensitisation from the strong stimulation of severe stress to visual areas. There is sufficient evidence to show the relationship between microglial activation and atrophy^25,46,47^, and microglial activation may influence brain morphological modification in these areas. On the other hand, Sinozaki *et al*. reported that activated microglia induced neuroprotective transformations of astrocytes after traumatic brain injury^48^. We therefore believe that there must be confounding factors, which decide whether glial function is protective or destructive. Further research should be conducted to identify these additional factors that decide whether glial function is either protective or destructive.

We observed a hypertrophic trend in the cluster extending from the medial part of the secondary visual cortex with moderate statistical threshold in VBM. Thus, we assume that severe stress might induce hypertrophy in this area. In this study, the morphological modification in the secondary visual cortex may be inconsistent. However, this is also similar to the case of a human study on spinal cord injury with pain recovery, in which brain atrophy and hypertrophy were neighbouring in the visual cortex^33^.

We did not detect significant atrophy in the amygdala; thus, we could not confirm the relationship between stress and regional brain morphological modification in the amygdala in humans. Although apoptosis induced by SPS in the amygdala has been previously reported^49–51^, our group demonstrated a significant increase of dendritic arborisation in the pyramidal neurons of basolateral amygdala 7 days after SPS^52^. Given these discrepant results in previous studies, some confounding factors may contribute to regional atrophy in the amygdala. However, the auditory fear conditioning paradigm enhances dendritic spine densities and alters the morphology of spines in the amygdala in mice^53^, and amygdalocortical pathways play important roles in fear conditioning^54^. In the case of humans, there are subcortical pathways: the ventral visual stream including the cortical-cortical long-range fasciculi from the visual cortex to the limbic areas^55^ including the amygdala. It has also been reported that the subcortical visual route to the amygdala relates to a fast response to fear stimuli^56^. We propose that the enhancement of projections and sensitisation from the amygdala to our obtained area in VBM after a severe stress situation should be analysed.

Our study did not detect atrophy in the hippocampus and we were not able to confirm the relationship between stress and atrophy in the hippocampus. Although some meta-analysis studies on hippocampal atrophy in PTSD have been reported, a recent meta-analysis suggested that hippocampal atrophy is not related to stress but related to disease^14^. On the other hand, enhancement of glucocorticoid receptor levels in the dorsal hippocampus of female rats that are induced by SPS has been reported^57^, and stress resilience brought about by sex differences may be a confounding factor for the relationship between stress and brain atrophy in the hippocampus. Further research to clarify the contribution of stress resilience arising from sex differences is needed.

Overall, the present study using VBM and immunohistochemistry has demonstrated that severe stress results in brain atrophy and microglial activation in the visual cortex and thalamus. Confirming a causal relationship between severe stress and PTSD is a major concern, and we consequently showed atrophy of the visual cortex and the thalamus as brain scars induced by severe stress. On the other hand, the results of this study are similar to the results of previous studies on chronic neuropathic pain, although we did not use the neuropathic pain paradigm. Pain-related areas could vary depending on psychological stress without pain itself. We believe that some sensitisation mechanism of severe stress would induce atrophy in the visual stream and that visual modulations might become potential interventions to stress-related diseases.

## Methods

### Stress model and tissue preparation

The SPS procedure was performed as per previously described methods^20,58^. The SPS protocol was based on a combined plural stress paradigm: immobilisation (compression with plastic bags) for 2 h, forced swimming for 20 min (24 °*C* ± 1 °*C*), and rest for 15 min, followed by drying and ether anaesthesia (until loss of consciousness), exposing completely within the 1^st^ day. Sprague Dawley (SD) male rats (Shimizu Laboratory Supplies Co., Ltd., Kyoto, Japan) were exposed to either SPS or sham stress (ether anaesthesia alone) at the age of 50 days and were then maintained in an undisturbed condition for a recovery period of 7 days (with two animals per cage). After the 7-day recovery period, the rats were perfused and fixed with 4% paraformaldehyde. We then removed the brains and skulls of the animals and immersed these structures in the same fixation medium for 3 days. Then, they were immersed into Fluorinert (3 M Japan Co., Tokyo, Japan) and stored until MRI acquisition. After MRI acquisition, some brains were used for immunohistochemistry. All experiments were approved by the Committee on Animal Research, Kyoto Prefectural University of Medicine, and were conducted according to the guidelines of the National Institutes of Health on animal care. We attempted to minimise the number of animals used and their suffering.

### *Ex vivo* brain MRI

*Ex vivo* brain MRI was performed using a 7 T Varian System (Agilent Technologies, Palo Alto, CA, USA). A Helmholtz small-volume coil (probe dimensions: 108/63 mm) was used for both radiofrequency excitation and signal detection. For segmentation purposes, a fast spin echo T2-weighted three-dimensional (3D) volume was collected (TR, 2000 ms; TE, 20 ms; FOV, 35 × 35 × 17.5 mm^3^; acquisition matrix, 128 × 128 × 64; zero filled to, 256 × 256 × 128; final voxel resolution, 0.137 × 0.137 × 0.137 mm^3^; pulse angle, 30°). Bias field inhomogeneity correction was applied to the acquired scans.

### In-house brain image template

We manufactured an in-house experimental T2-weighted image template using 8-week-old SD male rats (n = 9). For the application of script settings in the statistical parametric mapping software (SPM8, Wellcome Department of Cognitive Neurology, London, UK, http://www.fil.ion.ucl.ac.uk/spm/software/spm8/) used in the study, voxel size was magnified 10 times to adjust to the human brain size^59^. To increase signal-to-noise ratio as for a human VBM study^60^, the images were first de-noised by a 3D non-local means filter using an in-house C++ script with a general-purpose graphics processing units-based accelerating scheme. The images were coregistered to the in-house SD male rat template, created with six rat T2-weighted images by the sum-of-squared-differences minimisation algorithm and 12-parameter affine transformations according to a previously used method^61^. They were then segmented into images of grey matter, white matter, and cerebrospinal fluid by the unified segmentation method^62^ using the probabilistic maps of the SD rat brain by an in-house Matlab script according to the iterative expectation-maximisation strategy^63^. The obtained T2-weighted, grey matter, white matter, and cerebrospinal fluid images were used to create a customised, more population-specific template by the SPM8 tool for a diffeomorphic anatomical registration using exponentiated lie algebra (DARTEL) template creation^64^. DARTEL has been shown to produce a more accurate registration than the standard VBM procedure and is considered to be suitable for rat VBM^59^.

### Voxel-based morphometry

The experimental brain MR images were de-noised using a 3D non-local means filter after a 10-times voxel size magnification in the same way as described in the in-house brain image template section. The brain MR images were then coregistered and segmented into grey matter and spatially normalised using DARTEL in SPM8 with the in-house SD rat template and probabilistic maps. The processed grey matter images were then modulated using Jacobian determinants from the DARTEL procedure. The images were resampled into 1.5 × 1.5 × 1.5 mm^3^ (0.15 × 0.15 × 0.15 mm^3^) voxels and smoothed with an isotropic Gaussian kernel with an 8-mm (0.8 mm in original space) full width at half maximum. For a group-level statistical analysis, we performed the voxel-by-voxel two sample t-test across the whole brain, applied with the general linear model using SPM 8^65^. The cluster-defining height threshold was set at *p* < 0.001, with a determinant extent threshold at *p* modified with family-wise error (FWE) < 0.05.

### Immunohistochemistry

We performed immunohistochemistry of the ionised calcium-binding adaptor molecule 1 (Iba-1), an established molecular marker of activated microglia. The immunofluorescence procedure was previously described^20^. After the *ex vivo* MRI acquisition, the brains were randomly selected and sliced into 50-µm thick frontal frozen sections on a cryostat. Immunohistochemistry was applied to every fourth section (assuming a 50-μm thickness and 150 μm apart). We used rabbit anti-Iba-1 polyclonal antibody (019–19741, Wako, Osaka, Japan) as the primary antibody (immersed for 5 days at 4 °C) and Alexa Fluor 488 labelled goat anti-rabbit IgG (1:1000) (Molecular Probes, Eugene, OR, USA) as the secondary antibody (immersed for 2 days at 4 °C). Neurotrace 530/615 (Molecular Probes, Eugene, OR, USA) was applied as a counterstain to mark the neurons. Then, fluorescent images for obtained areas from VBM were collected using a confocal laser-scanning microscope (LSM; 510 Meta, Carl Zeiss, Germany).

### Morphometry and statistical analysis of the immunohistochemistry results

Morphometry was conducted using a computer image analysing system (Fiji)^66^ to analyse the average microglial number per slice and the average pixel size of the microglial cells. Five slices (best-stained) per sites were selected and used in this morphometry (numbers of rats; sham: n = 6, SPS: n = 5). For result reproducibility, an auto-threshold plugin (intermodal method)^67^ was used, and the cell cluster pixel size of the microglia was defined as >80 pixels for the thalamus and 39 pixels for visual cortex. We calculated mean deviations from the sham (%) ± standard error of means (SEM) for the microglial cell numbers and cell sizes and performed statistical analysis (one-tailed Welch’s t-test).

### Data availability

The data that support the findings of this study are available from the corresponding author upon reasonable request.

## Electronic supplementary material

Supplementary
