# Supplementary material for: Brain atrophy in the visual cortex and thalamus induced by severe stress in animal model
Source: Sci Rep. 2017 Oct 6;7:12731. doi: 10.1038/s41598-017-12917-z (PMC5630603; doi:10.1038/s41598-017-12917-z)
Supplement: Supplementary file 1 — Supplementary [file 41598_2017_12917_MOESM1_ESM.doc]

Supplementary Materials for

**Brain atrophy in the visual cortex and thalamus induced by severe stress**

**in animal model**

Takanobu Yoshii*, Naoya Oishi, Kazuya Ikoma, Isao Nishimura, Yuki Sakai, Kenichi Matsuda, Shunji Yamada, Masaki Tanaka, Mitsuhiro Kawata, Jin Narumoto, and

Kenji Fukui

*Correspondence to Takanobu Yoshii ([takanon@koto.kpu-m.ac.jp](mailto:takanon@koto.kpu-m.ac.jp))

This PDF file includes:

Supplementary figure 1 and its legend

Supplementary table

Supplementary figure 2 and its legend

**
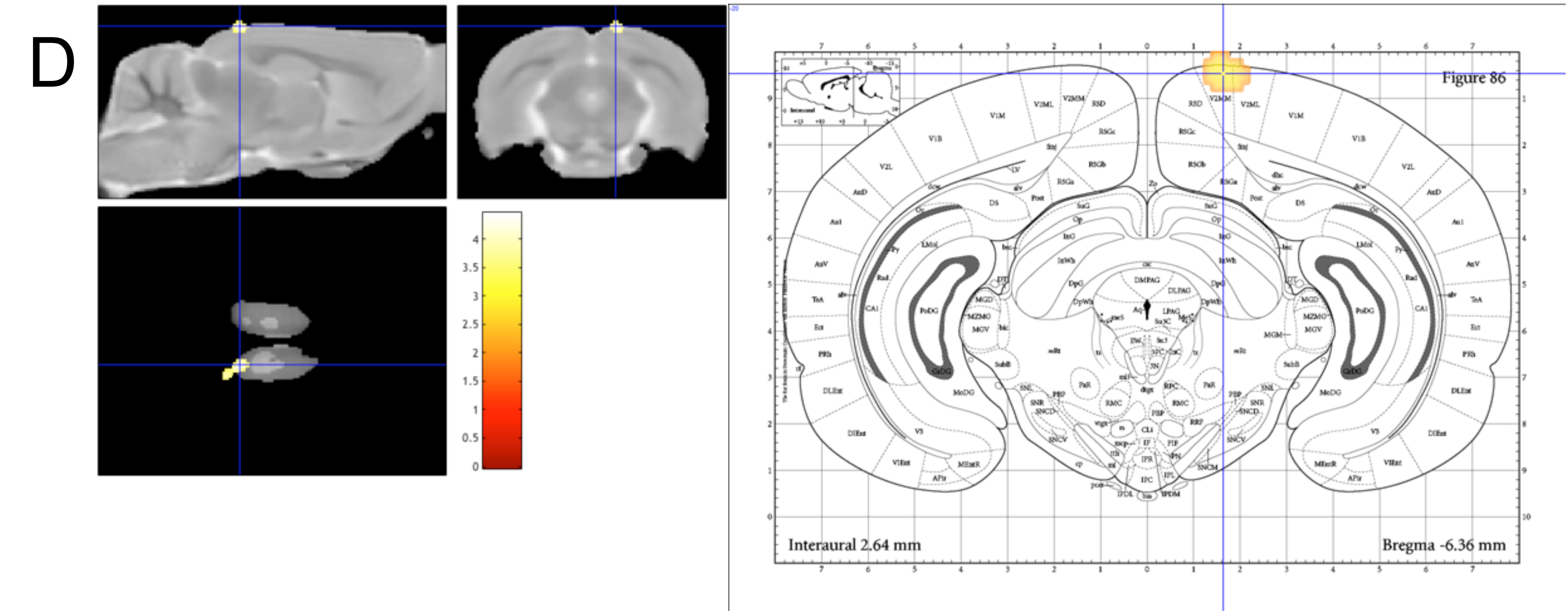
**

**Supplementary Figure 1**

**Results of global VBM detecting hypertrophy in 3D sections**

Three-dimensional brain section showing a trend of hypertrophy induced by SPS in the part of the secondary visual cortex (cluster D) (SPS, n = 18; sham, n = 17; *p* < 0.001, uncorrected). A coronal section with the peak coordinate piled upon the rat brain atlas^66^ is also presented. Colour bar units refer to t-scores. Cross hair lines indicate its peak coordinate, and it exists in the right secondary visual cortex, mediomedial area.

**Supplementary Table**

Hypertrophic cluster showing significant effects between the SPS and sham groups in voxel-based morphometry

|  | Cluster | Cluster | Peak | Peak | Peak |  |
| --- | --- | --- | --- | --- | --- | --- |
|  | *p* (FWE-corrected) | Cluster size (voxels) | T value | Z value | *p* (uncorrected) | Estimated area  (peak coordinate) |
| Cluster D | 0.780 | 189 | 4.43 | 3.89 | < 0.001 | Visual cortex  (rt V2MM) |

V2MM: secondary visual cortex, mediomedial area

**
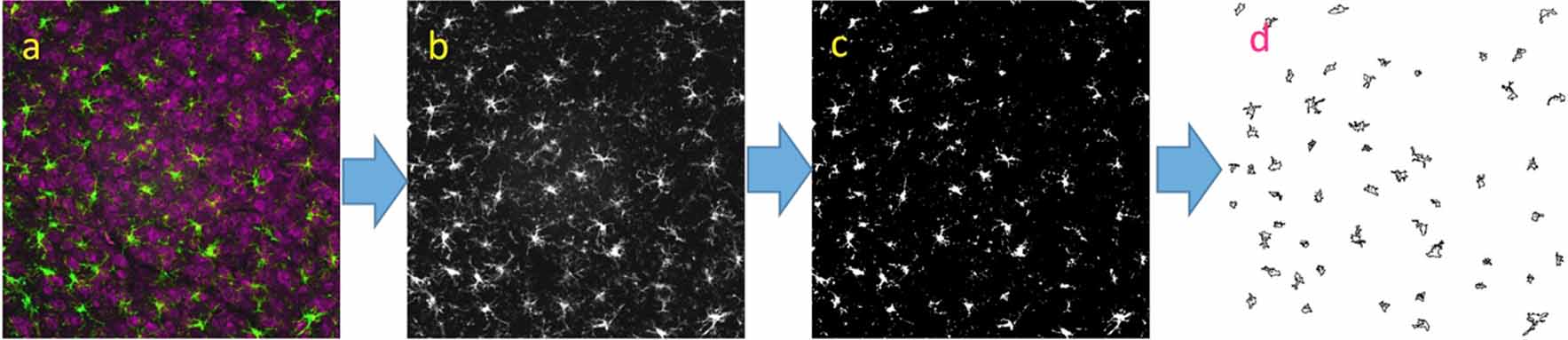
**

**Supplementary Figure 2**

**Morphometry course**

Morphometry course is presented; a: Raw data completely without modification; b: Colour-split with extraction only of green colour; c: Thresholded with Fiji software with an auto-threshold plugin, intermodal protocol applied; d: Outline of pixel count, which was secondarily thresholded with minimum cellular pixel numbers. A minimal cellular pixel number was defined as 80 pixels in the thalamus and 39 pixels in the visual cortex. Thirty-nine pixels indicate the calculated number, which was modified with the scale from the minimal cellular pixel number of the thalamus.
